# Volumetric Regression in Brain Metastases After Stereotactic Radiotherapy: Time Course, Predictors, and Significance

**DOI:** 10.3389/fonc.2020.590980

**Published:** 2021-01-08

**Authors:** Dominik Oft, Manuel Alexander Schmidt, Thomas Weissmann, Johannes Roesch, Veit Mengling, Siti Masitho, Christoph Bert, Sebastian Lettmaier, Benjamin Frey, Luitpold Valentin Distel, Rainer Fietkau, Florian Putz

**Affiliations:** ^1^ Department of Radiation Oncology, Universitätsklinikum Erlangen, Friedrich-Alexander-Universität Erlangen-Nürnberg, Erlangen, Germany; ^2^ Department of Neuroradiology, Universitätsklinikum Erlangen, Friedrich-Alexander-Universität Erlangen-Nürnberg, Erlangen, Germany

**Keywords:** brain metastases, stereotactic radiotherapy, stereotactic radiosurgery, volumetric analysis, MRI, longitudinal analysis, volumetric regression

## Abstract

**Background:**

There is insufficient understanding of the natural course of volumetric regression in brain metastases after stereotactic radiotherapy (SRT) and optimal volumetric criteria for the assessment of response and progression in radiotherapy clinical trials for brain metastases are currently unknown.

**Methods:**

Volumetric analysis *via* whole-tumor segmentation in contrast-enhanced 1 mm³-isotropic T1-Mprage sequences before SRT and during follow-up. A total of 3,145 MRI studies of 419 brain metastases from 189 patients were segmented. Progression was defined using a volumetric extension of the RANO-BM criteria. A subset of 205 metastases without progression/radionecrosis during their entire follow-up of at least 3 months was used to study the natural course of volumetric regression after SRT. Predictors for volumetric regression were investigated. A second subset of 179 metastases was used to investigate the prognostic significance of volumetric response at 3 months (defined as ≥20% and ≥65% volume reduction, respectively) for subsequent local control.

**Results:**

Median relative metastasis volume post-SRT was 66.9% at 6 weeks, 38.6% at 3 months, 17.7% at 6 months, 2.7% at 12 months and 0.0% at 24 months. Radioresistant histology and FSRT vs. SRS were associated with reduced tumor regression for all time points. In multivariate linear regression, radiosensitive histology (p=0.006) was the only significant predictor for metastasis regression at 3 months. Volumetric regression ≥20% at 3 months post-SRT was the only significant prognostic factor for subsequent control in multivariate analysis (HR 0.63, p=0.023), whereas regression ≥65% was no significant predictor.

**Conclusions:**

Volumetric regression post-SRT does not occur at a constant rate but is most pronounced in the first 6 weeks to 3 months. Despite decreasing over time, volumetric regression continues beyond 6 months post-radiotherapy and may lead to complete resolution of controlled lesions by 24 months. Radioresistant histology is associated with slower regression. We found that a cutoff of ≥20% regression for the volumetric definition of response at 3 months post-SRT was predictive for subsequent control whereas the currently proposed definition of ≥65% was not. These results have implications for standardized volumetric criteria in future radiotherapy trials for brain metastases.

## Introduction

Brain metastases are diagnosed in 170,000 patients annually in the United States and in 20% to 40% of patients with cancer (1). Despite their high prevalence, brain metastases are still underrepresented in clinical trials and basic scientific questions remain unanswered (2). Among others, there is currently insufficient knowledge on the natural course of volumetric regression in brain metastases following stereotactic radiotherapy (SRT) and diverse conceptions exist ranging from wax and wane type volume changes post-SRT to a continuous albeit slowed growth (3).

SRT is one of the most important treatment modalities for brain metastases today. Due to the continuous advancements in systemic treatments and consecutive improvements in extracranial control, more and more patients are treated with SRT during their illness (4). In the context of systemic treatments with extracranial long-term efficacy, sustained intracranial control becomes a necessary prerequisite for long-term survival. The significance of SRT is therefore expected to rise further and SRT needs to be optimized in terms of efficacy and tolerability due to continued research and clinical trials.

To enable further systematic progress, standardized criteria for the assessment of progression and response are of vital importance with volumetric analysis potentially being superior to traditional unidimensional measurements (5–8). The RANO-BM guideline is an important step in this direction. However, while the RANO-BM guideline stresses the importance of further research on volumetric assessment, it can only provide very incomplete guidance on volumetric criteria for the definition of response and progression due to a profound lack of scientific studies to base any recommendations upon (8). This is especially true for radiotherapy, where different criteria might be required than for systemic therapy trials. The basic understanding of the natural course of volumetric regression in brain metastases after stereotactic radiotherapy is currently incomplete and optimal criteria for the volumetric definition of response post-SRT are unknown (8).

In the present study we therefore sought to describe the natural course of volumetric regression of brain metastases after stereotactic radiotherapy in a large dataset of 419 brain metastases using 3145 whole-tumor segmentations. Predictors for volumetric regression in brain metastases were investigated. Wherever possible we adapted the RANO-BM recommendations or derived volumetric criteria from the established unidimensional RANO-BM criteria to support a standardized assessment of brain metastases. Furthermore, we evaluated the prognostic significance of volumetric response in brain metastases post-SRT for subsequent control by comparing the current RANO-BM recommendation to a commonly used lower volumetric threshold.

## Methods

### Ethics

Ethical review and approval was not required for this study in accordance with the local legislation and institutional requirements (BayKrG Art. 27). Written informed consent that data may be used for retrospective scientific studies was provided by the patients.

### Patient Population

We identified all patients who received stereotactic radiotherapy (SRT) for intracranial metastases at our institution between January of 2003 and April of 2015. From this group of 566 patients, patients were selected based on the following inclusion criteria: 1) stereotactic radiotherapy for intraparenchymal brain metastases from a solid cancer, 2) no prior SRT and no prior resection of the metastasis to be analyzed, 3) availability of contrast-enhanced T1-Mprage sequences with ≤ 1 mm slice thickness at baseline and at least once during follow-up. 419 brain metastases in 189 patients fulfilled these criteria and were selected for further analysis. Of these 189 patients, 97 were male (51.3%) and 92 were female (48.7%). Median age at start of radiotherapy was 62 years (range, 25–84 years).

In this cohort, the most common primary was malignant melanoma (42.5%, 178/419). 22.2% (93/419) of all metastases originated from lung cancer, 12.4% (52/419) from breast cancer and 10.5% (44/419) from renal cancer. Of the 93 metastases from lung cancer, 25.8% (24/93) were derived from small-cell lung cancer and the remaining 74.2% (69/93) from non-small-cell lung cancer. As common in brain metastases, melanoma, renal cell cancer and sarcoma were considered radioresistant histologies (9). Median pretreatment metastasis volume was 0.29 cm³, and median maximum diameter was 1.1 cm (**Table 1**).

**Table 1:** Characteristics of treated brain metastases (N = 419).

Metastasis characteristic	Total cohort (N = 419)	Subsets for analysis of	
		Regression in controlled metastases (N = 205)	Prognostic significance of volumetric regression (N = 179)
**Primary cancer histology, n (%)**			
Melanoma^R^	178 (42.5%)	78 (38.0%)	77 (43.0%)
Lung	93 (22.2%)	53 (25.9%)	39 (21.8%)
Breast	52 (12.4%)	28 (13.7%)	29 (16.2%)
Renal^R^	44 (10.5%)	23 (11.2%)	21 (11.7%)
Gastrointestinal	23 (5.5%)	14 (6.8%)	4 (2.2%)
Bladder/Urinary tract	6 (1.4%)	5 (2.4%)	6 (3.4%)
Sarcoma^R^	7 (1.7%)	2 (1.0%)	1 (0.6%)
Gynecologic	4 (1.0%)	1 (0.5%)	0 (0.0%)
Other	12 (2.9%)	1 (0.5%)	2 (1.1%)
**Pretreatment metastasis volume, cm³**			
Median (IQR)	0.29 (0.08–1.25)	0.33 (0.08–1.81)	0.26 (0.06–1.30)
**Pretreatment metastasis diameter, cm**			
Median (IQR)	1.1 (0.7–1.7)	1.1 (0.7–2.0)	1.1 (0.7–1.7)
**Pretreatment metastasis diameter, n (%)**			
< 1 cm	187 (44.6%)	89 (43.4%)	83 (46.4%)
1–2 cm	150 (35.8%)	66 (32.2%)	61 (34.1%)
2–3 cm	46 (11.0%)	27 (13.2%)	19 (10.6%)
> 3 cm	36 (8.6%)	23 (11.2%)	16 (8.9%)
**Upfront WBRT before SRT, n (%)**			
No	246 (58.7%)	109 (53.2%)	93 (52.0%)
Yes	173 (41.3%)	96 (46.8%)	86 (48.0%)
**Type of stereotactic radiotherapy, n (%)**			
Single session radiosurgery	215 (51.3%)	93 (45.4%)	85 (47.5%)
Fractionated stereotactic radiotherapy	204 (48.7%)	112 (54.6%)	94 (52.5%)
**SRS Single Dose, Gy**			
Median (IQR)	18.0 (18.0–20.0)	18.0 (18.0–20.0)	18.0 (16.0–20.0)
**FSRT: Single Dose, Gy**			
Median (IQR)	4.0 (3.0–4.0)	4.0 (3.0–4.0)	4.0 (3.0–4.0)
**FSRT: Total Dose, Gy**			
Median (IQR)	30.0 (20.0–40.0)	35.0 (28.0–40.0)	32.0 (20.0–40.0)
**Total BED_12-LQC_, Gy**			
Median (IQR)	52.4 (41.0–72.6)	55.6 (43.3–73.1)	55.6 (41.0–73.1)

IQR, interquartile range; WBRT, Whole-brain radiotherapy; BED_12-LQC_, Biologically effective dose for an alpha/beta ratio of 12, linear-quadratic-cubic model. Total BED_12-LQC_ was calculated including WBRT dose, if upfront WBRT was delivered prior to stereotactic radiotherapy.

^R^classified as radioresistant histology.

### Radiation Therapy

Patients received single-session radiosurgery (SRS) or fractionated stereotactic radiotherapy (FSRT) with a linear-accelerator based Novalis^®^ or Novalis-Tx^®^ system (BrainLAB, Feldkirchen, Germany). Patients were immobilized in an individually manufactured thermoplastic head mask attached to a stereotactic base frame (BrainLAB, Feldkirchen, Germany). Treatment planning was performed using Iplan (BrainLAB, Feldkirchen, Germany) (10, 11). Patients received a dedicated planning CT, which was rigidly coregistered with the baseline MRI using the Iplan software. The gross target volume (GTV) was delineated in the contrast-enhanced T1-Mprage sequence of the baseline MRI study. Planning target volume (PTV) was defined as GTV with an additional margin of 1–2 mm. During treatment, daily stereoscopic X-ray imaging (ExacTrac^®^) was used for setup verification and repositioning. For SRS, stereoscopic X-ray imaging was repeated after every couch rotation. 41.3% (173/419) of all metastases had been treated with upfront whole-brain radiotherapy (WBRT) before stereotactic radiotherapy (SRT) while 58.7% (246/419) received SRT alone. Median WBRT fraction dose was 3 Gy (interquartile range [IQR], 2–3 Gy) and median total WBRT dose was 40 Gy (IQR, 30–40 Gy). In case of upfront WBRT, WBRT was considered integral part of the treatment and the start date of WBRT was determined to be the start of radiotherapy for the respective brain metastases. In addition, WBRT dose was included in the calculation of the biologically effective dose (see below).

51.3% (215/419) of all metastases were treated with SRS while 48.7% (204/419) were treated with FSRT. Median single dose for SRS was 18 Gy. Different fractionation schemes were employed with FSRT. Median single dose for FSRT was 4 Gy and median total dose was 30 Gy (**Table 1**). As institutional policy smaller metastases were treated with SRS and larger metastases with FSRT. Median metastasis volume for SRS was 0.11 cm³ (IQR, 0.04–0.30 cm³) and median diameter was 0.8 cm (IQR, 0.6–1.1 cm) and median metastasis volume for FSRT was 1.19 cm³ (IQR, 0.31–4.28 cm³), with a median diameter of 1.7 cm (IQR, 1.1–2.4 cm).

As established by Wiggenraad et al., biologically effective dose (BED) was calculated based on an α/β ratio of 12 according to the LQC model (BED_12-LQC_) (12, 13):

$$BED_{12–LQC}=nd\;\left[1+\;\frac{d}{\left(\frac{\alpha }{\beta }\right)}-\frac{d^{2}}{\left(\frac{\alpha }{\gamma }\right)}\right]$$

With *n* being the number of fractions and *d* being the dose per fraction, *α/β* was assumed to be 12 Gy and α/γ 648 Gy² (12, 13). In case of upfront WBRT, BED_12-LQC_ were separately calculated for WBRT and SRT and added together to form the total BED_12-LQC_ used for further calculations.

### Follow-Up and Imaging

Images were collected on different Siemens 1.5 Tesla MRI scanners (Magnetom Aera or Magnetom Avanto) at our institution. All analyzed images consisted of 160 or 192 contiguous, sagittal, or transversal planes of 3-dimensional T1-weighted magnetization-prepared rapid gradient-echo images with 1 × 1 × 1 mm isotropic resolution (repetition time [TR] = 1,900 ms, echo time [TE] = 3.02 ms, inversion time [TI] = 1,100 ms, matrix = 256 × 265, field of view [FoV] = 250, flip angle = 15 degrees or TR = 2200 ms, TE = 2.67 ms, TI = 900 ms, matrix = 256 × 246, FoV = 250, flip angle = 8 degrees) after intravenous application of 0.2 ml/kg Dotarem (Guerbet) or 0.1 ml/kg Gadovist (Bayer), respectively.

Patients received MRI at baseline (median of 8 days before radiotherapy) and routinely at 6 weeks after stereotactic radiotherapy (SRT) and every 3 months thereafter. However, due to the retrospective nature of the study patients received MRI at slightly different points in time after SRT. To allow for analysis five time intervals were defined for volumetric measurements: 6 weeks after SRT = 6 ± 2 weeks after SRT, 3 months = 3 months ± 4 weeks after SRT, 6 months = 6 months ± 4 weeks after SRT, 12 months = 12 months ± 8 weeks after SRT and 24 months = 24 months ± 8 weeks after SRT.

### Volumetric Analysis

In total, 3,145 MRI studies were used for volumetric analysis (median of 6, IQR 4–9 per patient). Segmentation was performed using the open-source software 3D Slicer (version 4.5.0) (14). 3D Slicer is supported by the National Institutes of Health (NIH) and has a large worldwide developer community and adoption (15). The software offers different modules for segmentation, volume statistics and image coregistration. A custom-developed module was used that utilizes the built-in modules but accelerates the segmentation process by automating steps that do not require user interaction (16). Segmentation was performed semi-automatically using the VTK Fast Growcut method (17) as semiautomatic segmentation methods have been shown to decrease inter- and intra-observer variabilities (18, 19) and are much more time-efficient than manual delineation (20). Following a first semi-automatic segmentation step all segmentations were reviewed and corrected manually on a slice-by-slice basis using the editor module in 3D Slicer.

### Volumetric Extension of the RANO-BM Criteria for the Assessment of Progression Following SRT

We support the efforts for standardization in the assessment of response in brain metastases put forth by the RANO-BM working group (8). While the RANO-BM guideline stresses the importance of further research on volumetric analysis in brain metastases, the proposed criteria for volumetric analysis provided in the RANO-BM guideline are incomplete due to the lack of research supporting specific recommendations for volumetric assessment (8). We therefore adopted the basic concept from the RANO-BM guideline to derive volumetric criteria from the established unidimensional recommendations using spherical geometry. In this regard, the RANO-BM guideline recommends defining volumetric partial response as ≥ 65% reduction in volume (8). Following this principle, progression was defined as ≥ 72.8% increase in volume in the present study relative to nadir/baseline, which corresponds to a ≥ 20% increase in diameter of a perfect sphere (i.e., the unidimensional RANO-BM criteria for progression). In addition, as the RANO-BM guideline recommends to consider small brain metastases between 5 and 10 mm in diameter as unchanged unless the longest diameter changes by at least 3 mm, an additional absolute increase in volume of at least 0.2 cm³ was required for the definition of progression in the present study. This corresponds to the absolute volume increase of a 5 mm sphere growing by additional 3 mm in diameter. Due to this additional requirement and because the main aim of this study was to give an adequate representation of volumetric change in brain metastases following SRT, which are frequently < 5 mm in diameter, no lower size limit for brain metastases was defined in the present study. In addition, as SRT is a localized therapy, change in distant lesions, corticosteroid use or clinical status were not considered in the definition of progression in the present study. Lesions experiencing volumetric progression as per the criteria above but that subsequently showed spontaneous regression during imaging follow-up back to baseline/nadir volume or showed volumetric partial response as per the RANO-BM recommendation (i.e., ≥ 65% reduction in volume) were classified as pseudoprogression/radionecrosis instead of progression. Similarly, in the case of resection, metastases were classified as progression, radionecrosis or both based on histology (21).

### Statistical Analysis

For the analysis of the natural course of volumetric regression after SRT, only brain metastases were selected that did not show volumetric increase of ≥ 72.8% during their entire follow-up, did not receive resection for radionecrosis or progression and had a minimum imaging follow-up of at least 3 months. 205 metastases fulfilled these criteria and were used for this analysis. Of these, volumetric data was available for n=50 metastases at 6 weeks, n=166 metastases at 3 months, n=100 at 6 months, n=69 at 12 months and n=31 at 24 months.

Volumetric regression of brain metastases was compared between different groups using the Wilcoxon rank-sum test. Multiplicity adjustments were not performed, so p-values are descriptive and reflect a Type I error for the individual comparison. Univariate and multivariate linear regression were performed to evaluate potential predictors of residual relative metastasis volume at 3 months post-SRT.

For the evaluation of the prognostic significance of volumetric response at 3 months for subsequent local control, metastases were selected that had not progressed until then, had volumetric data available at 3 months post-SRT and had additional imaging follow-up. 179 brain metastases fulfilled these criteria and were used for this analysis. Time to local progression was calculated from the date of imaging 3 months post-SRT until progression as per the criteria defined above or cases were censored at the date of last imaging follow-up. Local control was compared between brain metastases with and without volumetric response (defined as ≥ 20% and ≥ 65% volume reduction, respectively) by means of the Kaplan-Meier method and the logrank test. Furthermore, the prognostic significance of volumetric response and other prognostic factors at 3 months post-SRT for subsequent local control was evaluated in univariate and multivariate Cox’s regression analysis.

Covariates were included in multivariate models based on biologic considerations. P-values < 0.05 were considered statistically significant. All statistical analyses were performed using IBM SPSS 21.

## Results

### Volumetric Regression in the Entire Cohort of Brain Metastases

**Figure 1:**
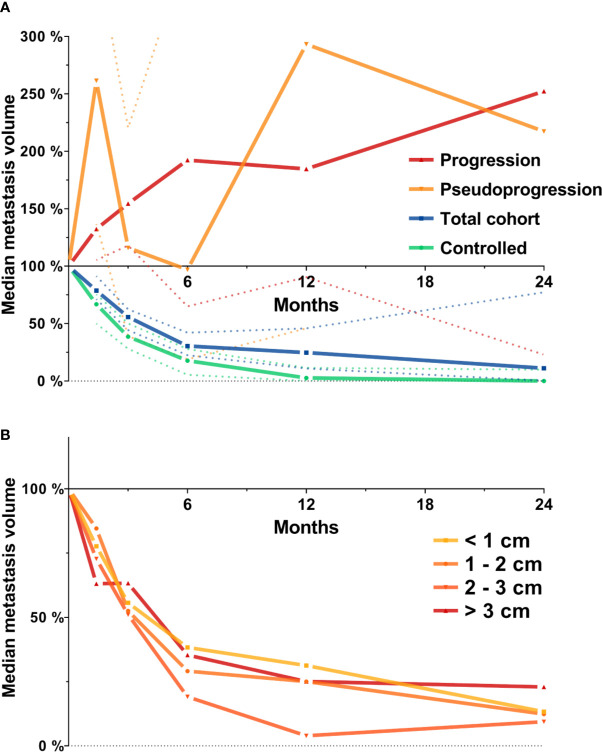
Median metastasis volume over time following stereotactic radiotherapy in the entire cohort of metastases (N = 419). Tumor volumes are expressed relative to baseline volume. **(A)** Median metastasis volume over time for the total cohort (N = 419, blue), for lesions experiencing progression (N = 78, red) or pseudoprogression during follow-up (N = 16, orange) and for the subset of controlled metastases used for further analyses (N = 205, see methods section for definition, green). Dotted lines represent the 95% confidence interval. **(B)** Median metastasis volume over time in the entire cohort stratified by baseline metastasis diameter.

First, we investigated the course of volumetric regression in the entire cohort of 419 brain metastases. In the entire cohort, median relative metastasis volume following stereotactic radiotherapy was 78.7% at 6 weeks, 55.8% at 3 months, 30.4% at 6 months, 24.7% at 12 months, and 11.2% at 24 months (**Figure 1A**). We also assessed volumetric regression stratified by metastasis diameter. Interestingly, even though the number of metastasis > 2 cm was limited (n = 82, **Table 1**), the observed time course of volumetric regression was quite similar for metastases < 1 cm, between 1 and 2 cm, between 2 and 3 cm and for lesions > 3 cm (**Figure 1B**). Seventy-eight metastases experienced progression during follow-up. These progressive lesions showed an almost continuous increase in median relative metastasis volume (132.3% at 6 weeks, 154.5% at 3 months, 192.3% at 6 months, 184.6% at 12 months, and 252.3% at 24 months post-SRT). Interestingly, metastases experiencing pseudoprogression (n = 16) during follow-up, i.e., volumetric progression followed by spontaneous regression in size, had two peaks in median relative metastasis volume at 6 weeks and at 12 months post-SRT (261.4% at 6 weeks, 116.0% at 3 months, 96.9% at 6 months, 293.2% at 12 months, and 217.4% at 24 months post-SRT, **Figure 1A**).

### Natural Course of Volumetric Regression in Controlled Brain Metastases

**Figure 2:**
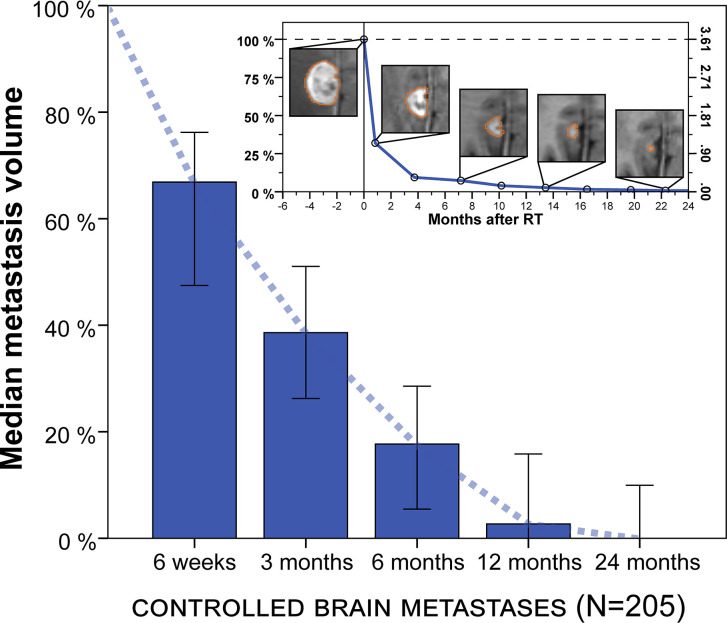
Median metastasis volume over time following stereotactic radiotherapy in controlled brain metastases (N = 205). Tumor volumes are expressed relative to baseline volume. Error bars show the 95% confidence interval. Note: Volumetric regression is most pronounced in the first 3 months but continues thereafter. Upper right inset: Example of longitudinal volumetry in a larger brain metastasis treated with fractionated stereotactic radiotherapy (FSRT). Left ordinate shows relative tumor volume and right ordinate shows absolute metastasis volume (cm³). Segmentation is shown for different measurement time points.

The natural course of volumetric regression was investigated in 205 lesions that did not develop progression or radionecrosis during their entire imaging follow-up of at least 3 months (see methods section) to avoid superposition by progressive or pseudoprogressive lesions in different phases of growth and to obtain a reference for physiologic volume changes post-SRT. Median relative metastasis volume following stereotactic radiotherapy was 66.9% at 6 weeks (IQR, 23.0%–87.2%, n = 50), 38.6% at 3 months (IQR, 8.1%–71.1%, n = 166), 17.7% at 6 months (IQR, 0.0%–43.9%, n = 100), 2.7% at 12 months (IQR, 0.0%–30.9%, n = 69) and 0.0% at 24 months (IQR, 0.0%–18.9%, n = 31) (**Figure 2**). Similar results were obtained in a sensitivity analysis when excluding the minority of lesions from small-cell lung cancer (median relative tumor volume, 6 weeks: 67.3%, 3 months: 40.0%, 6 months: 21.7%, 12 months: 4.5%, 24 months: 0.0%).

**Figure 3:**
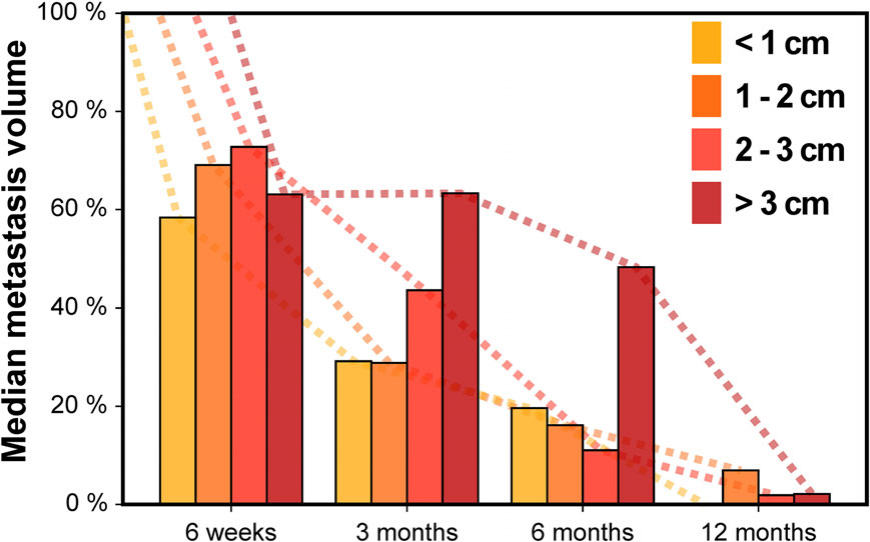
Median metastasis volume over time following stereotactic radiotherapy in controlled brain metastases stratified by baseline metastasis diameter.

Next, we assessed the impact of tumor size on volumetric regression stratifying by metastasis diameter (**Figure 3**). For metastases < 1 cm, median tumor volume was 58.4% at 6 weeks, 29.2% at 3 months, 19.6% at 6 months and 0.0% at 12 months. For tumors 1–2 cm, median volume was 69.1% at 6 weeks, 28.8% at 3 months, 16.2% at 6 months and 6.9% at 12 months. In the subgroup of metastases with 2–3 cm, median tumor volume was 72.8% at 6 weeks, 43.6% at 3 months, 11.1% at 6 months and 1.9% at 12 months. Finally, for brain metastases > 3 cm, median tumor volume was 63.1% at 6 weeks, 63.3% at 3 months, 48.2% at 6 months but 2.2% at 12 months. Interestingly, across all tumor size categories metastasis regression was not significantly different for any of the time points studied, despite differences in volumetric regression tended towards being significant at 3 months post-SRT (Kruskal-Wallis p = 0.052). In addition, when dichotomizing metastases according to tumor diameter, there was no significant difference in volumetric regression between lesions < 1 cm and ≥ 1 cm for any of the time points studied (Wilcoxon rank-sum p ≥ 0.070). Similarly, no significant difference in volumetric regression was observed for metastases with 2–3 cm diameter compared to lesions < 2 cm (Wilcoxon rank-sum p ≥ 0.226). Only for brain metastases ≥ 3 cm, median volumetric regression was diminished at 3 and 6 months and volumetric regression at 3 months was significantly lower than for metastases < 3 cm (p = 0.015). Despite the number of larger metastases was limited (**Table 1**), we thus found no indication that the course of volumetric regression post-SRT differed fundamentally in relation to tumor size for brain metastases up to 3 cm in diameter.

**Figure 4:**
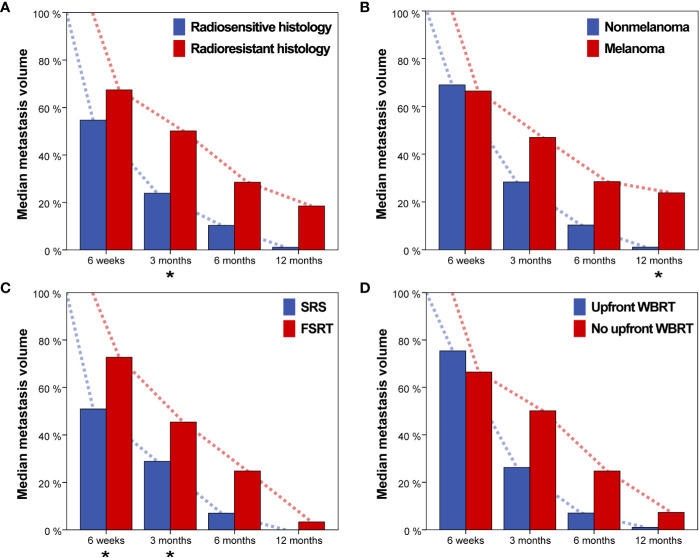
Median metastasis volume over time for **(A)** radiosensitive vs. radioresistant histology (i.e., melanoma, renal cell carcinoma or sarcoma), **(B)** Nonmelanoma vs. melanoma histology, **(C)** single-session radiosurgery (SRS) vs. fractionated stereotactic radiotherapy (FSRT), and **(D)** Upfront whole-brain radiotherapy (WBRT) vs. no upfront WBRT. Asterisks indicate significant intergroup differences for the respective timepoint.

Brain metastasis regression over time was compared for primary tumor histology, SRS vs. FSRT, upfront Whole-brain radiotherapy (WBRT) vs. no upfront WBRT and for melanoma vs. nonmelanoma histology (**Figure 4**). Radioresistant histology (i.e., melanoma, sarcoma, and renal cell carcinoma) was associated with reduced median tumor regression for all time points (median relative tumor volume, 6 weeks: 67.4% vs. 54.7%, 3 months: 50.1% vs. 23.9%, 6 months: 28.6% vs. 10.3% and 12 months: 18.5% vs. 1.1%). Difference in tumor regression for radioresistant and radiosensitive histology was significant at 3 months (p = 0.015, Wilcoxon rank-sum test, **Figure 4A**). As melanoma was the most common histology in this series, we additionally compared volumetric regression for metastases with melanoma and nonmelanoma histology. Melanoma brain metastases showed reduced median tumor regression at 3, 6, and 12 months post-SRT (median relative tumor volume, 6 weeks: 66.5% vs. 69.1%, 3 months: 47.0% vs. 28.4%, 6 months: 28.6% vs. 10.3% and 12 months: 23.8% vs. 1.1%) with the difference at 12 months being significant (p = 0.019, **Figure 4B**). SRS was associated with more profound median tumor regression for all time points in comparison to FSRT (median relative tumor volume, 6 weeks: 50.9% vs. 72.8%, 3 months: 28.9% vs. 45.4%, 6 months: 7.0% vs. 24.8% and 12 months: 0.0% vs. 3.3%). Differences were significant for 6 weeks and 3 months (p = 0.030 and p = 0.020, respectively, Wilcoxon rank-sum test, **Figure 4C**). For brain metastases treated with upfront WBRT before SRT, we observed reduced median volumetric regression at 6 weeks but increased volumetric regression at all other time points. None of these differences was significant however (median relative tumor volume, 6 weeks: 75.4% vs. 66.5%, 3 months: 26.3% vs. 50.1%, 6 months: 7.0% vs. 24.8%, 12 months: 1.1% vs. 7.3%, **Figure 4D**).

To better understand which treatment and tumor-related factors determine volumetric regression of brain metastases, we investigated which parameters influence relative metastasis volume at 3 months post-SRT in linear regression analysis. In univariate analysis, radioresistant tumor histology (p = 0.011), FSRT vs. SRS (p = 0.048) and increasing pretreatment metastasis volume (p = 0.032) were significant factors for worse tumor regression. In multivariate analysis, radioresistant histology (p = 0.006) remained the only significant predictor for reduced metastasis regression at 3 months post-SRT (**Table 2**).

**Table 2:** Predictive factors for residual relative metastasis volume at 3 months in linear regression analysis (N = 166).

Parameter	Univariate		Multivariate	
	β coefficient (95% CI)	*p*-value	β coefficient (95% CI)	*p*-value
**Primary tumor histology,** **radioresistant vs. radiosensitive**	**0.153 (0.036**–**0.271)**	**0.011**	**0.171 (0.050**–**0.292)**	**0.006**
SRS vs. FSRT	**-0.120 (-0.239**–**0.001)**	**0.048**	-0.122 (-0.251–0.007)	0.064
Pretreatment metastasis volume, cm³	**0.008 (0.001**–**0.016)**	**0.032**	0.006 (-0.002–0.014)	0.143
Total BED_12-LQC_, Gy	-0.002 (-0.006–0.001)	0.217	-0.001 (-0.008–0.006)	0.764
Upfront WBRT	-0.103 (-0.222–0.015)	0.088	-0.019 (-0.231–0.192)	0.856

Univariate and multivariate linear regression for relative metastasis volume at 3 months normalized to baseline tumor volume. SRS, Stereotactic radiosurgery; FSRT, Fractionated stereotactic radiotherapy; BED_12-LQC_, Biologically effective dose for an alpha/beta ratio of 12, linear-quadratic-cubic model; WBRT, Whole-brain radiotherapy.

Significant covariates are highlighted in bold.

### Prognostic Significance of Volumetric Response at 3 Months Post-SRT for Subsequent Local Control

**Figure 5:**
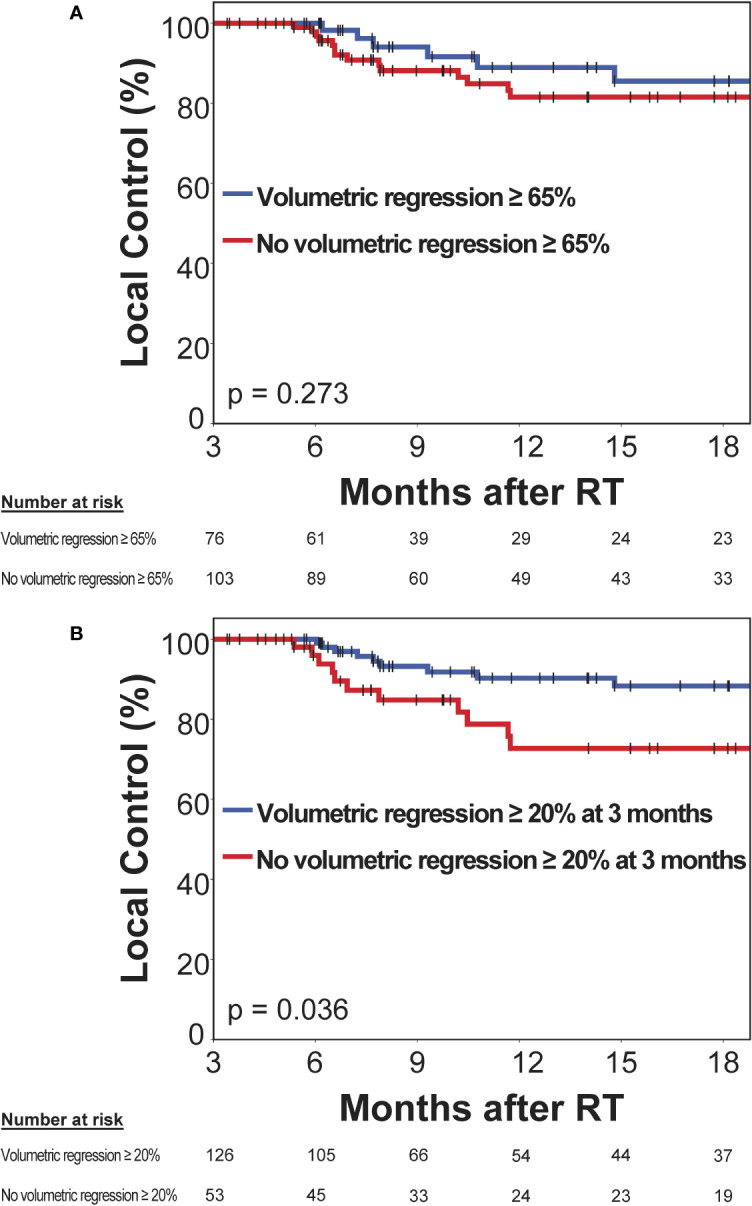
Prognostic significance of volumetric response at 3 months post-stereotactic radiotherapy (SRT) for subsequent local control in a second subset without prior progression and subsequent imaging follow-up. Kaplan-Meier plots for two different thresholds for the definition of volumetric response are shown: **(A)** ≥ 65% as per the current RANO-BM recommendation and **(B)** ≥ 20%, which is commonly considered the lowest volumetric threshold that can be reliably detected. Vertical bars represent censored cases.

Next, we evaluated the prognostic significance of volumetric response at 3 months following stereotactic radiotherapy for subsequent local control of irradiated brain metastases. A second subset of 179 brain metastases was used to evaluate the prognostic significance of volumetric response for subsequent local control, in which imaging was performed at 3 months post-SRT and that had not progressed until then. The RANO-BM criteria currently recommend defining partial response volumetrically as a reduction in tumor volume of at least 65% (8). The minimum volume reduction that can be reliably detected, however, is commonly considered to be as low as 20% (22, 23). As it is currently unclear, which volume cut-off is superior for the volumetric definition of partial response (8), we evaluated both thresholds in their ability to differentiate low- from high-risk metastases during subsequent follow-up. At 3 months post-SRT, volumetric response as defined by a volumetric reduction of ≥ 65% relative to baseline did not significantly differentiate metastases that subsequently developed progression and those that remained subsequently controlled (median not reached, 1-year local control [15 months post-SRT]: 81.5% vs. 85.5%, logrank p = 0.273) (**Figure 5A**). Moreover, in multivariate analysis, when including type of stereotactic radiotherapy, pretreatment metastasis volume, primary tumor histology, upfront WBRT and BED_12-LQC_, volumetric reduction ≥ 65% did not significantly discriminate between metastases with subsequent control and those developing progression (HR 0.79, p = 0.290). In contrast, volumetric regression ≥ 20% compared to baseline did significantly differentiate high-risk metastases from those with subsequent local control (median not reached, 1-year local control [15 months post-SRT] 72.7% vs. 88.3%, logrank p = 0.036) (**Figure 5B**). This was equally observed in a sensitivity analysis when excluding the minority of metastases from small-cell lung cancer (median not reached, 1-year local control [15 months post-SRT] 72.7% vs. 87.4%, logrank p = 0.038). When examining the most common histology, melanoma brain metastases alone, volumetric regression ≥ 20% also separated high- from low-risk metastases (1-year local control [15 months post-SRT]: 78.6% vs. 92.9%) but significance was lost (p = 0.204) in the context of reduced statistical power (n = 77 vs. 179 metastases). In multivariate analysis across all histologies, when including type of stereotactic radiotherapy, pretreatment metastasis volume, primary tumor histology (i.e., radiosensitive vs radioresistant histology), BED_12-LQC_ and the use of upfront WBRT, volumetric regression ≥ 20% at 3 months was the only significant predictor for local control during the subsequent follow-up period (HR 0.40, p = 0.023) (**Table 3**). Interestingly, volumetric regression at 3 months was also predictive for subsequent local control when assessed as continuous parameter in univariate (HR 0.9996 per percent decrease in volume, p = 0.003) and in multivariate analysis (HR 0.9996 per percent decrease in volume, p = 0.010).

**Table 3:** Predictive value of volumetric response at 3 months post-radiotherapy for local control during subsequent follow-up: Univariate and multivariate Cox’s regression analysis (N = 179).

Parameter	Univariate		Multivariate	
	HR (95% CI)	*p*-value	HR (95% CI)	*p*-value
**Volumetric regression ≥ 20%**	**0.45 (0.21**–**0.97)**	**0.041**	**0.40 (0.18**–**0.88)**	**0.023**
Volumetric regression ≥ 65%	0.63 (0.28–1.44)	0.277	Not included	Not included
SRS vs. FSRT	0.55 (0.25–1.23)	0.148	0.50 (0.19–1.30)	0.153
Pretreatment metastasis volume, cm³	1.03 (0.99–1.07)	0.101	1.02 (0.98–1.06)	0.311
Primary tumor histology, radioresistant vs. radiosensitive	0.81 (0.38–1.73)	0.581	0.68 (0.29–1.59)	0.374
Upfront Whole-brain radiotherapy	0.49 (0.22–1.10)	0.082	0.64 (0.18–2.33)	0.503
BED_12-LQC_, Gy	0.98 (0.95–1.01)	0.123	0.99 (0.94–1.03)	0.584

Volumetric response was defined as ≥ 20% and ≥ 65% volume reduction relative to baseline, respectively. Volumetric regression ≥ 65% was not a significant predictor when included in the multivariate model in place of volumetric regression ≥ 20% (HR 0.79, p = 0.290).

HR, Hazard ratio; WBRT, Whole-brain radiotherapy; FSRT, Fractionated stereotactic radiotherapy; SRS, Stereotactic radiosurgery; BED_12-LQC_, Biologically effective dose for an alpha/beta ratio of 12, linear-quadratic-cubic model.

Significant covariates are highlighted in bold.

## Discussion

Several attempts have been undertaken in the past to describe volume changes in brain metastases after stereotactic radiotherapy (SRT) (3, 24–29). However, although these studies provided important evidence, they were limited by a low number of analyzed metastases (25, 29, 30), few time points studied (24), or the fact that volumetric measurements were only carried out heuristically (3, 26) and not by means of whole-tumor segmentation.

In every case, analyses were not restricted to controlled brain metastases, so that progressing metastases in different stages of growth impeded the accurate assessment of volumetric regression over time.

In the present study, we attempted to overcome these limitations by only including metastases that had no evidence of progression or radionecrosis during follow-up. Volumetric criteria for progression and radionecrosis were objective and derived from the RANO-BM guideline by following the overarching concepts of the guideline (8).

Furthermore, we used volumetric data from 3145 whole-tumor segmentations defined in high resolution 1-mm³ isotropic T1-MPrage sequences for the present study but limited the analysis to five time-points post-SRT where enough volumetric data was available. In addition, in the present analysis, we studied median relative metastasis volume that is less sensitive to outliers than the mean, that has been used in past studies (27). Overall, the present study might provide the most comprehensive picture of volumetric regression in brain metastases following SRT to date. Important findings are that volumetric regression in brain metastases post-SRT does not occur at a constant rate but is most pronounced in the first 6 weeks to 3 months. Despite decreasing over time, volumetric regression continues beyond 6 months post-SRT.

Multiple clinical studies in the past have assumed a linear reduction in relative tumor volume over time, which may lead to wrong conclusions. For example, a recent retrospective study on the prognostic significance of volumetric regression in melanoma brain metastases counterintuitively found a worse overall survival and a higher rate of distant brain metastases in cases with faster volumetric regression post-SRT. The authors had assumed a continuous rate of volumetric reduction and calculated a “tumor dynamic index” as average percentage decrease in metastasis volume per day (31). As follow-up imaging was done 1–3 months post-SRS, a higher average percentage decrease in volume could have simply reflected an earlier follow-up MRI due to new neurologic symptoms or overall worsening patient condition (31).

Importantly, we also discovered that controlled brain metastases showed complete resolution during long-term follow-up, while the main clinical aim of stereotactic radiotherapy is of course to enable long-term control and to support prolonged survival. This finding emphasizes that stereotactic radiotherapy is a highly effective treatment modality and that complete resolution of imaging findings during follow-up is expected and does not à posteriori invalidate the imaging diagnosis of brain metastasis. Finally, a description of the natural course of volumetric regression in brain metastases may constitute an important reference when developing criteria for the volumetric assessment of brain metastases in clinical trials. We investigated different predictors for volumetric regression. In the final multivariate analysis radioresistant histology was the only significant predictor for reduced volumetric regression at 3 months post-SRT. While this finding has been described before (24, 27), we were able to confirm it in a large dataset accounting for possible confounders in multivariate analysis.

This finding might reflect fundamental radiobiologic differences in brain metastases according to primary histology. Individualizing dose prescription and fractionation schemes in brain metastases according to histology could represent an important approach to further improve the efficacy and tolerability of stereotactic radiotherapy.

In addition, the finding of differential response to stereotactic radiotherapy according to histology also highlights that brain metastases constitute a heterogenous disease - a fact that has contributed to the underrepresentation of brain metastases in clinical trials and lack of research. In the present study, we deliberately did not attempt to limit the analysis to a homogeneous subgroup instead we attempted to describe volumetric regression in a continuous cohort of brain metastases that is more representative of the heterogeneity found in daily clinical practice.

Standardized criteria for the assessment of response and progression are however of vital importance and the RANO-BM guideline represents a major advancement in this regard (8). Beside stressing the importance of more research on the volumetric assessment of brain metastases, the RANO-BM guideline discusses two thresholds for the volumetric definition of partial response, notably ≥ 20% and ≥ 65% volume reduction (8). A threshold of ≥ 65% volume reduction is derived from the established unidimensional criterion of ≥ 30% decrease in diameter (i.e., a sphere decreasing by 30% in diameter decreases by ca. 65% in volume) and therefore represents a continuation of the current unidimensional criteria. However, a threshold of ≥ 20% volume reduction, which is commonly considered to be the lowest threshold that can be reliably detected (22, 23), may have a higher sensitivity in detecting metastases with a favorable prognosis. In the present study we compared both thresholds in their ability to discriminate between metastases with subsequent control and those developing progression at 3 months post-SRT. Volumetric regression ≥ 20% at 3 months post-SRT significantly predicted subsequent control and was the only significant prognostic factor for subsequent local control in multivariate analysis, whereas volumetric regression ≥ 65% did not significantly differentiate metastases that subsequently developed progression and those that remained subsequently controlled.

These findings suggest that – in the context of stereotactic radiotherapy – ≥ 20% volume reduction could be a better threshold for the volumetric definition of response. Further studies will need to confirm this finding, however. Moreover, different criteria for the definition of response may be needed in the context of stereotactic radiotherapy in comparison to systemic therapy trials.

Volumetric analysis has many methodologic advantages, including the more reliable measurement of complex lesions (5, 6), the invariance to different scan planes and patient positioning and generally that it allows to reliably detect smaller changes in tumor size than unidimensional assessment (7, 8).

Already in 2001, Sorenson et al. showed in a JCO publication that volumetry using whole-tumor segmentation in 219 glioblastoma cases lead to reduced inter- and intrareader variability compared to measuring three orthogonal diameters. Furthermore, they observed differences in response classification in more than every fourth patient (6).

The fact that, nearly two decades later, volumetric analysis is still not standard in clinical trials today, cannot be explained by a lack of supporting research alone. Instead, for the most time, volumetric assessment has been very costly and time-consuming (8). Whereas Sorenson et al. still needed to scan physical films for subsequent slice-by-slice segmentation (6), semiautomatic techniques have emerged that are much more time-efficient and reduce inter- and intra-observer variability (18–20). Recently, the advent of artificial neural networks has even enabled accurate fully automatic segmentation of brain tumors (32, 33). Moreover, radiomic analyses also necessarily require tumor segmentations and are increasingly incorporated into clinical trials (34). It is therefore very likely that volumetric assessment will ultimately become the new standard for the assessment of response and progression in clinical trials. As stereotactic radiotherapy will have an increasingly important role to play in enabling intracranial long-term control, further research on volumetric changes and on optimized criteria for the volumetric assessment of progression and response is much-needed.

### Limitations

As this was a retrospective study, the timing of imaging studies was not strictly standardized and we were limited to study time points, where enough volumetric data was available. Also, fewer imaging studies were available for later time points. Standardization in treatment benefited from the fact that all imaging and treatment was done at a single institution. However, due to the retrospective nature it cannot be excluded that treatment-related factors could have been influenced by hidden confounders. Similarly, as selection of metastases for single-session radiosurgery vs. fractionated stereotactic radiotherapy was dependent on tumor size, no definitive conclusions in regard to differences in volumetric regression between these two modalities should be drawn. As most metastases in this series were ≤ 2 cm in diameter, generalizability of the results to large metastases may be limited.

## Conclusion

Volumetric regression of brain metastases after SRT does not occur at a constant rate. Instead, volumetric regression is most pronounced in the first 3 months. Despite decreasing over time, volumetric regression continues beyond 6 months post-SRT and may lead to complete resolution of controlled lesions by 24 months. Radioresistant histology is associated with slower regression, which might reflect fundamental radiobiologic differences. Volumetric analysis may have a role in identifying metastases at risk for subsequent progression. A lower threshold of ≥ 20% for the definition of volumetric response post-SRT was superior to the current RANO-BM recommendation of ≥ 65% in this study. Further volumetric studies in brain metastases after stereotactic radiotherapy are of high importance to establish volumetric criteria for standardized assessment in clinical trials.

## Data Availability Statement

The raw data supporting the conclusions of this article will be made available by the authors, without undue reservation.

## Ethics Statement

Ethical review and approval was not required for the study on human participants in accordance with the local legislation and institutional requirements. The patients/participants provided their written informed consent to participate in this study.

## Author Contributions

DO, MS, SL, LD, RF, and FP conceptualized the manuscript. DO, MS, and FP investigated the findings. DO and FP performed the analysis. MS, VM, SM, CB, SL, BF, LD, RF, and FP provided the resources. DO, MS, TW, JR, VM, SM, CB, SL, BF, LD, RF, and FP performed the writing. CB, BF, LD, RF, and FP supervised the findings. All authors contributed to the article and approved the submitted version.

## Funding

FP was supported by a grant from the Interdisciplinary Center for Clinical Research (IZKF) Erlangen: rotation program for physician scientists (https://www.izkf.med.fau.de/).

## Conflict of Interest

The authors declare that the research was conducted in the absence of any commercial or financial relationships that could be construed as a potential conflict of interest.

## References

[1] Tabouret E Chinot O Metellus P Tallet A Viens P Goncalves A Recent trends in epidemiology of brain metastases: an overview. Anticancer Res (2012) 32(11):4655–62. 23155227

[2] Preusser M Weller M Brain metastasis research: a late awakening. Chin Clin Oncol (2015) 4(2):17. 10.3978/j.issn.2304-3865.2015.05.01 26112803

[3] Patel TR McHugh BJ Bi WL Minja FJ Knisely JP Chiang VL A comprehensive review of MR imaging changes following radiosurgery to 500 brain metastases. AJNR Am J Neuroradiol (2011) 32(10):1885–92. 10.3174/ajnr.A2668 PMC7966021 21920854

[4] Badiyan SN Regine WF Mehta M Stereotactic Radiosurgery for Treatment of Brain Metastases. J Oncol Pract (2016) 12(8):703–12. 10.1200/JOP.2016.012922 27511715

[5] Follwell MJ Khu KJ Cheng L Xu W Mikulis DJ Millar BA Volume specific response criteria for brain metastases following salvage stereotactic radiosurgery and associated predictors of response. Acta Oncol (2012) 51(5):629–35. 10.3109/0284186x.2012.681066 22537310

[6] Sorensen AG Patel S Harmath C Bridges S Synnott J Sievers A Comparison of diameter and perimeter methods for tumor volume calculation. J Clin Oncol (2001) 19(2):551–7. 10.1200/jco.2001.19.2.551 11208850

[7] Bauknecht HC Romano VC Rogalla P Klingebiel R Wolf C Bornemann L Intra- and interobserver variability of linear and volumetric measurements of brain metastases using contrast-enhanced magnetic resonance imaging. Invest Radiol (2010) 45(1):49–56. 10.1097/RLI.0b013e3181c02ed5 19996757

[8] Lin NU Lee EQ Aoyama H Barani IJ Barboriak DP Baumert BG Response assessment criteria for brain metastases: proposal from the RANO group. Lancet Oncol (2015) 16(6):e270–8. 10.1016/S1470-2045(15)70057-4 26065612

[9] Brown PD Brown CA Pollock BE Gorman DA Foote RL Stereotactic radiosurgery for patients with “radioresistant” brain metastases. Neurosurgery (2002) 51(3):656–65; discussion 65-7. 10.1097/00006123-200209000-00009 12188943

[10] Koca S Distel L Lubgan D Weissmann T Lambrecht U Lang-Welzenbach M Time course of pain response and toxicity after whole-nerve-encompassing LINAC-based stereotactic radiosurgery for trigeminal neuralgia-a prospective observational study. Strahlentherapie und Onkol Organ der Deutschen Rontgengesellschaft [et al] (2019) 195(8):745–55. 10.1007/s00066-019-01450-9 30877350

[11] Putz F Muller J Wimmer C Goerig N Knippen S Iro H Stereotactic radiotherapy of vestibular schwannoma: Hearing preservation, vestibular function, and local control following primary and salvage radiotherapy. Strahlentherapie und Onkol Organ der Deutschen Rontgengesellschaft [et al] (2017) 193(3):200–12. 10.1007/s00066-016-1086-5 27928625

[12] Wiggenraad R Verbeek-de Kanter A Kal HB Taphoorn M Vissers T Struikmans H Dose-effect relation in stereotactic radiotherapy for brain metastases. A systematic review. Radiother Oncol (2011) 98(3):292–7. 10.1016/j.radonc.2011.01.011 21316787

[13] Joiner Quantifying cell kill and survival. In: vdKA JM, editor. Basic clinical radiobiology. London, England: Hodder Arnold (2009).

[14] Fedorov A Beichel R Kalpathy-Cramer J Finet J Fillion-Robin JC Pujol S 3D Slicer as an image computing platform for the Quantitative Imaging Network. Magn resonance Imaging (2012) 30(9):1323–41. 10.1016/j.mri.2012.05.001 PMC3466397 22770690

[15] Pinter C Lasso A Wang A Jaffray D Fichtinger G SlicerRT: Radiation therapy research toolkit for 3D Slicer. Med Phys (2012) 39(10):6332–8. 10.1118/1.4754659 23039669

[16] Putz F Knippen S Fietkau R Eine semiautomatische Segmentierungspipeline zur sequentiellen Volumetrie von intrakraniellen Tumoren. Strahlentherapie und Onkol Organ der Deutschen Rontgengesellschaft [et al] (2017) 193(S1):1–194. 10.1007/s00066-017-1137-6

[17] Zhu L Kolesov I Gao Y Kikinis R Tannenbaum A eds. An Effective Interactive Medical Image Segmentation Method using Fast GrowCut. In: Int Conf Med Image Comput Comput Assist Interv Workshop on Interactive Methods. Boston, MA (2014).

[18] Zhao B Tan Y Tsai WY Qi J Xie C Lu L Reproducibility of radiomics for deciphering tumor phenotype with imaging. Sci Rep (2016) 6(1):23428. 10.1038/srep23428 27009765 PMC4806325

[19] Balagurunathan Y Kumar V Gu Y Kim J Wang H Liu Y Test-retest reproducibility analysis of lung CT image features. J Digital Imaging (2014) 27(6):805–23. 10.1007/s10278-014-9716-x PMC4391075 24990346

[20] Odland A Server A Saxhaug C Breivik B Groote R Vardal J Volumetric glioma quantification: comparison of manual and semi-automatic tumor segmentation for the quantification of tumor growth. Acta Radiol (2015) 56(11):1396–403. 10.1177/0284185114554822 25338837

[21] Shaw E Scott C Souhami L Dinapoli R Kline R Loeffler J Single dose radiosurgical treatment of recurrent previously irradiated primary brain tumors and brain metastases: final report of RTOG protocol 90-05. Int J Radiat Oncol Biol Phys (2000) 47(2):291–8. 10.1016/s0360-3016(99)00507-6 10802351

[22] Yang DY Sheehan J Liu YS ChangLai SP Pan HC Chen CJ Analysis of factors associated with volumetric data errors in gamma knife radiosurgery. Stereotactic Funct Neurosurg (2009) 87(1):1–7. 10.1159/000177622 19039257

[23] van de Langenberg R de Bondt BJ Nelemans PJ Baumert BG Stokroos RJ Follow-up assessment of vestibular schwannomas: volume quantification versus two-dimensional measurements. Neuroradiology (2009) 51(8):517–24. 10.1007/s00234-009-0529-4 PMC2710491 19418046

[24] Da Silva AN Nagayama K Schlesinger D Sheehan JP Early brain tumor metastasis reduction following Gamma Knife surgery. J Neurosurg (2009) 110(3):547–52. 10.3171/2008.4.17537 18821832

[25] Hawighorst H Essig M Debus J Knopp MV Engenhart-Cabilic R Schonberg SO Serial MR imaging of intracranial metastases after radiosurgery. Magn resonance Imaging (1997) 15(10):1121–32. 10.1016/s0730-725x(97)00178-1 9408133

[26] Huber PE Hawighorst H Fuss M van Kaick G Wannenmacher MF Debus J Transient enlargement of contrast uptake on MRI after linear accelerator (linac) stereotactic radiosurgery for brain metastases. Int J Radiat Oncol Biol Phys (2001) 49(5):1339–49. 10.1016/S0360-3016(00)01511-X 11286842

[27] Iyer A Harrison G Kano H Weiner GM Luther N Niranjan A Volumetric response to radiosurgery for brain metastasis varies by cell of origin. J Neurosurg (2014) 121(3):564–9. 10.3171/2014.4.Jns131502 24878286

[28] Pan HC Sheehan J Stroila M Steiner M Steiner L Gamma knife surgery for brain metastases from lung cancer. J Neurosurg (2005) 102(Suppl):128–33. 10.3171/jns.2005.102.s_supplement.0128 15662795

[29] Sharpton SR Oermann EK Moore DT Schreiber E Hoffman R Morris DE The volumetric response of brain metastases after stereotactic radiosurgery and its post-treatment implications. Neurosurgery (2014) 74(1):9–15; discussion 6; quiz 6. 10.1227/neu.0000000000000190 24077581

[30] Kim WH Kim DG Han JH Paek SH Chung HT Park CK Early significant tumor volume reduction after radiosurgery in brain metastases from renal cell carcinoma results in long-term survival. Int J Radiat Oncol Biol Phys (2012) 82(5):1749–55. 10.1016/j.ijrobp.2011.03.044 21640509

[31] Zubatkina I Ivanov P Early imaging radioresponsiveness of melanoma brain metastases as a predictor of patient prognosis. J Neurosurg (2018) 129(2):354–65. 10.3171/2017.1.Jns162075 28841116

[32] Kamnitsas K Ledig C Newcombe VFJ Simpson JP Kane AD Menon DK Efficient multi-scale 3D CNN with fully connected CRF for accurate brain lesion segmentation. Med Image Anal (2017) 36:61–78. 10.1016/j.media.2016.10.004 27865153

[33] Xue J Wang B Ming Y Liu X Jiang Z Wang C Deep-learning-based Detection and Segmentation-assisted Management on Brain Metastases. Neuro-oncology (2019) 22(4):505–14. 10.1093/neuonc/noz234 PMC7158643 31867599

[34] Aerts HJ Velazquez ER Leijenaar RT Parmar C Grossmann P Carvalho S Decoding tumour phenotype by noninvasive imaging using a quantitative radiomics approach. Nat Commun (2014) 5:4006. 10.1038/ncomms5006 24892406 PMC4059926

